# Might proton pump or sodium‐hydrogen exchanger inhibitors be of value to ameliorate SARs‐CoV‐2 pathophysiology?

**DOI:** 10.14814/phy2.14649

**Published:** 2020-12-25

**Authors:** Kirk P. Conrad

**Affiliations:** ^1^ Departments of Physiology and Functional Genomics, and of Obstetrics and Gynecology University of Florida College of Medicine Gainesville FL USA

**Keywords:** COVID‐19, endosomal complexes required for transport, endosomal pH, exosomes, extracellular vesicles, viroporin

## Abstract

Discovering therapeutics for COVID‐19 is a priority. Besides high‐throughput screening of compounds, candidates might be identified based on their known mechanisms of action and current understanding of the SARs‐CoV‐2 life cycle. Using this approach, proton pump (PPIs) and sodium‐hydrogen exchanger inhibitors (NHEIs) emerged, because of their potential to inhibit the release of extracellular vesicles (EVs; exosomes and/or microvesicles) that could promote disease progression, and to directly disrupt SARs‐CoV‐2 pathogenesis. If EVs exacerbate SARs‐CoV‐2 infection as suggested for other viruses, then inhibiting EV release by PPIs/NHEIs should be beneficial. Mechanisms underlying inhibition of EV release by these drugs remain uncertain, but may involve perturbing endosomal pH especially of multivesicular bodies where intraluminal vesicles (nascent exosomes) are formed. Additionally, PPIs might inhibit the endosomal sorting complex for transport machinery involved in EV biogenesis. Through perturbing endocytic vesicle pH, PPIs/NHEIs could also impede cleavage of SARs‐CoV‐2 spike protein by cathepsins necessary for viral fusion with the endosomal membrane. Although pulmonary epithelial cells may rely mainly on plasma membrane serine protease TMPRSS2 for cell entry, PPIs/NHEIs might be efficacious in ACE2‐expressing cells where viral endocytosis is the major or a contributing entry pathway. These pharmaceutics might also perturb pH in the endoplasmic reticulum‐Golgi intermediate and Golgi compartments, thereby potentially disrupting viral assembly and glycosylation of spike protein/ACE2, respectively. A caveat, however, is that facilitation not inhibition of avian infectious bronchitis CoV pathogenesis was reported in one study after increasing Golgi pH. Envelope protein‐derived viroporins contributed to pulmonary edema formation in mice infected with SARs‐CoV. If similar pathogenesis occurs with SARs‐CoV‐2, then blocking these channels with NHEIs could ameliorate disease pathogenesis. To ascertain their potential efficacy, PPIs/NHEIs need evaluation in cell and animal models at various phases of SARs‐CoV‐2 infection. If they prove to be therapeutic, the greatest benefit might be realized with the administration before the onset of severe cytokine release syndrome.

## Graphical abstract

This work presents the pros and cons for considering proton pump and sodium‐hydrogen exchanger inhibitors as potential therapeutics in COVID‐19.

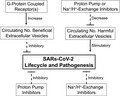

## 1 INTRODUCTION

The world currently faces a coronavirus pandemic, which started in 2019 (COVID‐19). Humans are naïve to the inciting virus, SARS‐CoV‐2, a highly infectious enveloped RNA virus especially virulent in individuals with co‐morbidities, although anyone can be at risk for severe disease and death. It is still early in the pandemic for formulating definitive statistics, but one recent CDC report for the United States covering January 22‐May 30, 2020 included 1,320,488 laboratory‐confirmed COVID‐19 patients. It was estimated that, when considering people of all ages and both sexes, approximately 14.0% required hospitalization, 2.3% was admitted to the ICU, and 5.4% died (Stokes et al., 2020). One of the severest manifestations was respiratory distress syndrome requiring mechanical ventilation. Morbidity and mortality increased with the age of affected individuals, disproportionately affected Hispanic, Black and American Indian or Alaska Natives, as well as those with pre‐existing medical conditions such as chronic lung or cardiovascular disease (Stokes et al., 2020).

At the start of a pandemic, it is difficult to ascertain an accurate R0, which reflects the average number of people infected by one person who harbors the virus, but for an estimate of ~3, in order for “herd immunity” to occur approximately 70% of the US population or 230 million people will ultimately need to be infected (Metcalf et al.,) Clearly, the human cost of attaining “herd immunity” would be extremely high (Pollan et al., 2020). Thus, all the current preventative policies including “social distancing” are imperative and need to be exercised for the foreseeable future in the US and around the world until safe and effective vaccine(s) are produced and widely distributed, in order to reduce the morbidity and mortality, and to prevent large cohorts of people from becoming sick at once, which would overwhelm health care personnel and infrastructure. The extent of “lock down” will necessarily vary over time as it needs to be periodically ramped up and then down on a recurring basis to balance human and economic health. Of additional concern is that many individuals are SARS‐CoV‐2 positive, but asymptomatic and shedding virus (Stokes et al., 2020; Sun & Weng, 2020). Finally, the degree to which or length of time someone who was infected will be immune to future exposure is uncertain. On balance, there remains much to be learned about the epidemiology, natural history, and pathogenesis of SARS‐CoV‐2 infection, as well as the immunological response to the virus.

Nevertheless, given the potentially long timeline for development, production and distribution of an effective vaccine for the entire global population, therapeutics are desperately needed in the meantime to ameliorate COVID‐19 pathology, and reduce severe morbidity and mortality. To this end, it is vital to determine whether therapeutics currently in use for other disease entities might be efficacious, thereby allowing their off‐label application for treatment of COVID‐19. Initial interrogation of large database(s) of electronic medical records may be useful, in order to ascertain whether patients taking medications commonly prescribed for other medical conditions experienced a less severe clinical course of SARs‐CoV‐2 infection. Finally, identifying potential therapeutics based on their mechanism of action in preclinical studies might accelerate their development as therapeutics for treating SARs‐CoV‐2 infection.

With a paucity of potential therapeutics, at least to date, in addition to high throughput screening of large repositories of small molecules and other approaches in the laboratory, inductive reasoning may reveal potential therapeutics based on a priori knowledge of COVID‐19 pathogenesis and the mechanism of action of existing pharmaceutics. Through this approach, two classes of drugs that might prove efficacious are the proton pump inhibitors (PPIs) and sodium‐hydrogen exchanger inhibitors (NHEIs). The general rationale for considering these inhibitors as potential therapeutics is based on their ability to impair the release of extracellular vesicles (EVs; exosomes and/or microvesicles), because EVs may facilitate disease progression, and their potential to disrupt the life cycle and directly inhibit pathogenic mechanisms of SAR‐CoV‐2. The pros and cons of considering PPIs and NHEIs for treatment of COVID‐19, as well as the uncertainties, in part due to insufficient knowledge of SARs‐CoV‐2 biology, are presented in the following sections.

It should be acknowledged at the outset that the ideas expressed herein arose from inductive reasoning based on a priori knowledge of enveloped RNA viruses, EVs, and the mechanisms of action of PPIs and NHEIs, all for the most part as revealed by others. Nevertheless, the purpose of this *Review* is twofold: (a) to advance the idea for consideration and scrutiny by the scientific and medical communities that PPIs and NHEIs may have therapeutic potential in treating SARs‐CoV‐2; and (b) attempt a critical and balanced evaluation of the pros and cons of PPIs and NHEIs as potential therapeutics including discussion of the possible mechanisms. It should be noted, of course, that Remdesivir, Dexamethasone, and Famotidine have recently emerged as potentially promising therapeutic candidates in clinical studies (Beigel et al., 2020; Freedberg et al., 2020; Horby et al., 2020).

## 2 RATIONALE

### 2.1 Enveloped RNA viruses and extracellular vesicles share structural and functional features

Interestingly, enveloped RNA viruses and EVs may have one or more of the following attributes in common: (i) small size of ~100 nm, (ii) shared host pathways of biogenesis, (iii) similar cargo including RNA, (iv) capacity to fuse with cell membranes of target cells, and (v) transfer of cargo to recipient cells that, in turn, affects cellular function (Mathieu et al., 2019; Nolte‐'t Hoen et al., 2016). Of course, one major difference is that EVs unlike viruses do not replicate. As pointed out by others, it remains unclear whether EVs or enveloped viruses may have first emerged in evolution, that is, enveloped viruses may have co‐opted the biogenesis pathways of EVs leading to the envelopment of genetic material in a lipid bilayer containing proteins that permit cell targeting and infection, or EVs may have arisen from enveloped viruses deficient in replicative potential (Margolis & Sadovsky, 2019; Nolte‐'t Hoen et al., 2016). Finally, an important, though perhaps obvious caveat which nevertheless deserves mentioning here, is that not all enveloped RNA viruses are the same. Specifically, there are major and nuanced differences in their life cycles, which mean that biological mechanisms learned about one RNA virus, like HIV‐1, may not necessarily apply to another, for example, SARs‐CoV‐2.

### 2.2 Extracellular vesicles

#### 2.2.1 EVs modify viral transmission from infected to uninfected cells

Considerable evidence supports the concept that EVs released from virally infected cells may abet or impede the infection of naive cells (Nolte‐'t Hoen et al., 2016; Raab‐Traub & Dittmer, 2017). As examples, EVs may display viral antigens on their surface, thereby serving as decoy targets for host antibodies, whereas other EVs may contain miRNAs from virus‐resistant cells that confer protection in susceptible cells (Delorme‐Axford et al., 2013; Nolte‐'t Hoen et al., 2016). The net balance of EV actions in a virally infected individual, whether facilitatory or inhibitory of infection, may be difficult to predict, and perhaps also depends on the phase of infection, for example, incubation, prodromal, early versus late symptomatic, or convalescence. This uncertainty pertains to SARs‐CoV‐2, but if the role of EVs in propagating infection predominates, then reducing the release of EVs should be therapeutic. On the other hand, if the predominant role of EVs is to inhibit infection, then increasing the release of EVs could prove beneficial. Of course, the best strategy would be to decrease those EVs containing cargo that promote infection, and increase EVs harboring cargo which impede infection.

#### 2.2.2 PPIs inhibit EV release

Proton pump inhibitors inhibited EV release by tumor cells, one consequence being enhanced cellular retention and activity of chemotherapeutic agents by reducing EV‐mediated efflux (Chalmin et al., 2010; Federici et al., 2014; Guan et al., 2017). Mice bearing tumors produced by injection of human melanoma or mouse colon carcinoma cells showed significant reduction in plasma EVs after treatment with PPIs (Chalmin et al., 2010; Federici et al., 2014). Crucial to the potential therapeutic role of PPIs in SARs‐CoV‐2 infection through inhibition of EV release is the assumption that PPIs can inhibit EV release from *nontransformed* cells, which apparently has not been investigated. Indeed, if PPIs do not inhibit EV release from nontransformed cells such as pulmonary epithelial cells, then repurposing of PPIs for treatment of SARs‐CoV‐2 at least through an EV‐dependent mechanism is moot. Nevertheless, what is known about the mechanism(s) for decreased EV release by PPIs in transformed cells, although incompletely understood, merits discussion.

#### 2.2.3 Possible mechanism for PPI inhibition of EV release: blockade of vacuolar‐type H^+^‐ATPase

By inhibiting vacuolar‐type H^+^‐ATPase (V‐H^+^‐ATPase), PPIs raised intraluminal pH of intracellular organelles in the endocytic pathway (at least as documented in lysosomes) (Luciani et al., 2004). In contrast, bafilomycin, another inhibitor of V‐H^+^‐ATPase, also raised intraluminal pH in lysosomes (Christensen et al., 2002), but rather than reducing EV release like PPIs, bafilomycin increased EV release (Savina et al., 2003). Similarly, (hydroxy)chloroquine, a weak base that accumulates in acidic compartments including endocytic vesicles and lysosomes, thereby buffering H^+^ and raising intraluminal pH (Al‐Bari, 2017; Chiang et al., 1996; Maxfield, 1982; Ohkuma & Poole, 1978), also increased EV release (Savina et al., 2003). In the case of bafilomycin and (hydroxy)chloroquine, the mechanism proposed for increased EV release by Savina and colleagues was calcium efflux from endosomes into cytosol that, in turn, stimulated calcium‐dependent mechanisms of EV release (Beraldo et al., 2001; Christensen et al., 2002; van Niel et al., 2018; Savina et al., 2003, 2005). However, the exact transport mechanism(s) that mediated calcium efflux from endosomes in response to intraluminal alkalinization was not unequivocally identified.

One possible explanation for the apparent paradox that, on the one hand, reduced intraluminal acidification after V‐H^+^‐ATPase inhibition by PPIs was associated with *decreased* EV release, whereas on the other, reduced intraluminal acidification after V‐H^+^‐ATPase inhibition by bafilomycin or (hydroxy)chloroquine was associated with *increased* EV release, may relate to timing. That is, in the shorter term, trafficking of already formed endosomal multivesicular bodies (MVBs) containing intraluminal vesicles (ILVs; nascent exosomes, a subclass of EVs) to the plasma membrane where they subsequently fuse and release exosomes may be accelerated by calcium‐dependent mechanisms as discussed earlier. In the longer term, however, because V‐H^+^‐ATPase mediates the acidification of organelles including the ER, Golgi, trans‐Golgi network, endocytic vesicles, early endosomes, and MVBs (Brown et al., 1983; Demaurex et al., 1998; Kellokumpu, 2019; Maeda & Kinoshita, 2010; Mellman et al., 1986; Scott & Gruenberg, 2011), inhibition of V‐H^+^‐ATPase by PPIs and other agents that impair acidification may disrupt biological processes in the endocytic pathway including maturation of early to late endosomes and formation of ILVs within MVBs (Brown et al., 1983; Falguieres et al., 2008; Kellokumpu, 2019; Maeda & Kinoshita, 2010; Matsuo et al., 2004; Scott & Gruenberg, 2011) (although there is not complete agreement (Huotari & Helenius, 2011; Vacarri et al., 2010)). Thus, the release of EVs is decreased, perhaps even in the face of increased cytosolic calcium, because ILVs are not being formed in the MVB. To begin addressing these potential explanations, time course experiments of the effects of V‐H^+^‐ATPase inhibition on EV release might be instructive.

Another potential explanation for decreased EV release by PPIs is that, rather than increasing calcium release as proposed by Savina et al. (2003), by raising endosomal pH, PPIs actually reduce calcium efflux from endosomes. In other words, endosomal calcium concentration decreases as endosomes acidify (Gerasimenko et al., 1998; Petersen et al., 2020). Although this scenario is not consistent with the findings of Savina et al. for bafilomycin and chloroquine, there appears to be inconsistency between these publications in regards to the relationship between endosomal [H^+^] and calcium concentrations. Perhaps this relationship could be different in different endosomal compartments? Finally, in addition to V‐H^+^‐ATPase, PPIs could conceivably inhibit the function of other endosomal proteins by covalently binding to thiol groups, ultimately reducing exosome biogenesis (Liu et al., 2013).

#### 2.2.4 Alternative mechanism for PPI inhibition of EV release: blockade of endosomal sorting complex for transport machinery

Another potential explanation for the apparent paradox raised above is that PPIs decreased the release of EVs through another mechanism independent of organelle intraluminal pH. That is, disruption of endosomal (or microvesicle) pathways, and ultimately, of EV release by PPIs perhaps occurred through interference with the assembly of the endosomal sorting complex for transport (ESCRT) proteins at the MVB‐limiting membrane critical to protein sorting, budding, and release (scission) of ILVs into the MVB lumen. In light of the reverse topology of ILVs budding into the MVB, cytoplasmic proteins like dynamin do not have access to the outside of the membrane neck to effect membrane scission. Therefore, ILV budding occurs through the recruitment of cytosolic ESCRT proteins, which regulate the sorting of ubiquitinated proteins, membrane budding, and scission (Hurley, 2015; Rossman & Lamb, 2013). Similarly, the ESCRT proteins have been implicated in the budding and scission of microvesicles at the plasma membrane (Hurley, 2015).

A brief overview of ESCRT biology follows. The ubiquitin‐interacting motif (UIM) of hepatocyte growth factor‐regulated tyrosine kinase substrate, Hrs, a protein in the ESCRT‐0 complex, binds to mono‐ubiquitin moieties of proteins in the limiting membrane of early endosomes, which are destined to be sorted into ILVs (Bishop et al., 2002; Polo et al., 2002). Interestingly, the Hrs UIM is itself mono‐ubiquitinated (Polo et al., 2002). The clathrin‐box motif and FYVE domains of Hrs interact with clathrin and phosphatidylinositol 3‐phosphate, respectively, which leads to enrichment of Hrs within microdomains of the early endosomal‐limiting membrane (Raiborg et al., 2001, 2006). Subsequently, a PSAP motif in Hrs binds to the ubiquitin E2 variant (UEV) domain of tumor susceptibility gene 101 (TSG101), a protein in the ESCRT‐1 complex, thereby recruiting ESCRT‐1 to the early endosomal‐limiting membrane (Lu et al., 2003; Pornillos et al., 2002, 2003), where it also interacts with the mono‐ubiquitinated proteins (Bishop et al., 2002). Finally, ESCRT‐I recruits ESCRT‐II, that in turn mobilizes ESCRT‐III, which is the critical ESCRT mediating membrane scission (Hurley & Hanson, 2010; Katzmann et al., 2002). An ESCRT‐associated pathway involving syndecan‐syntenin‐Alix (ALG‐2 interacting protein X) interaction has more recently been shown to be involved in ILV biogenesis (Baietti et al., 2012). Finally, there are ESCRT‐independent mechanisms, too (Hessvik & Llorente, 2018).

As presented in greater detail later (see section on *Viruses* below), the retrovirus HIV‐1 (and other RNA viruses) harnesses the ESCRT machinery for viral assembly and cellular egress through the interaction of the UEV domain in TSG101 with a PTAP motif in the HIV‐1 Gag‐C terminal p6 domain (Hurley & Cada, 2018). Budding of viruses assembled at the plasma membrane into the extracellular space is comparable to the membrane topology of ILVs budding into the MVB or microvesicles budding from the plasma membrane, insofar as cytoplasmic proteins like dynamin do not have access to the outside of the bud neck to carry out membrane scission (Rossman & Lamb, 2013). Therefore, HIV‐1 like ILV membrane scission occurs through the recruitment of ESCRT proteins, that is, HIV‐1 co‐opts the host ESCRT machinery (Hurley & Cada, 2018; Rossman & Lamb, 2013).

Of note, Tjandra, Carter, and colleagues reported that, after intracellular activation of the prodrug, PPIs covalently bound to cysteine 73 in the ubiquitin‐binding pocket of TSG101, which they speculated might impede TSG101 interaction with a ubiquitin moiety in HIV‐1 Gag‐C terminal p6 protein at the plasma cell membrane, thereby inhibiting subsequent viral assembly and budding. Because viral production was arrested earlier at the budding rather than scission stage as observed with disruption of the UEV domain in TSG101 with a PTAP motif in the HIV‐1 Gag‐C terminal p6 domain, the authors further suggested a chaperone function for TSG101 in the budding process (Strickland et al., 2017). PPI inhibition of viral assembly was prevented by a C73A substitution in TSG101 that precluded binding with PPIs. Interestingly, however, PPIs did not interfere with the interaction of the Gag C‐terminal p6 PTAP motif with the TSG101 UEV domain. Nevertheless, by impeding the HIV‐1 Gag‐C terminal p6 ubiquitin—TSG101 interaction, TSG101 did not accumulate on the plasma membrane, the site of viral assembly and budding (Strickland et al., 2017). The authors concluded that PPI disruption of the TSG101 UEV domain—HIV‐1 Gag‐C terminal p6 ubiquitin moiety prevented the recruitment of TSG101, and also impaired viral assembly at an earlier stage than disruption of the TSG101 UEV domain—Gag C‐terminal p6 PTAP domain (Strickland et al., 2017).

By analogy, because the UIM motif of Hrs is also mono‐ubiquitinated and the UEV domain of TSG101 can bind to mono‐ubiquitinated proteins, another potential interaction between Hrs and TSG101, in addition to that mediated by the Hrs PSAP motif, might be through ubiquitin (Pornillos et al., 2002). Indeed, the binding affinity of TSG101 with HIV‐1 Gag‐C terminal p6 region was greater for p6 fused to ubiquitin than to either p6 or ubiquitin alone, suggesting cooperatively between the p6 PTAP motif and p6 ubiquitin in binding to TSG101 (Garrus et al., 2001; Pornillos et al., 2002). Thus, it is not inconceivable that by disrupting the interaction between mono‐ubiquinated Hrs and the UEV domain of TSG101, PPIs could reduce the binding affinity of the TSG101‐Hrs interaction, an important step in ILV biogenesis which could ultimately reduce exosome release.

However, a strong argument against this possibility is that, although the HIV‐1 Gag‐C p6 PTAP‐TSG101 UEV and Hrs PSAP‐TSG101 UEV interactions may be analogous, Pornillos and colleagues showed that ubiquitin‐binding mutations in the TSG101 UEV had no effect on Hrs‐TSG101 interaction (Pornillos et al., 2003). This finding contrasts with the interaction of ubiquitin in the HIV‐1 Gag‐C terminal p6 region with the TSG101 UEV domain that confers increased binding affinity as discussed earlier (Garrus et al., 2001; Pornillos et al., 2002). Moreover, Tjandra, Carter, and coworkers further showed that at the concentration of 50 µM, tenatoprazole did not inhibit ligand‐induced epidermal growth factor receptor downregulation, which generally entails dissociation of ligand from receptor in the acidic early endosome, binding of ubiquitinated EGFR with the UIM domain of Hrs, and of the PSAP motif in Hrs with the ubiquitin E2 variant (UEV) domain of TSG101, ultimately leading to trafficking of the EGFR from the early to late endosomes and lysosomes where they are degraded (Lu et al., 2003; Strickland et al., 2017). Nor did tenatoprazole interfere with localization of TSG101 to the midbody of cells ultimately required for cytokinesis. On the other hand, in the same publication, Tjandra, Carter, and coworkers reported that tenatoprazole did inhibit constitutive recycling of EGFR that was not bound to its ligand confirming a role for TSG101 in this physiological process susceptible to PPI inhibition (Strickland et al., 2017). Nevertheless, because PPIs inhibited exosome release (Chalmin et al., 2010; Federici et al., 2014; Guan et al., 2017), it might be worthwhile to exclude the possibility (though admittedly remote based on the insightful work of Pornillos and colleagues, and Tjandra, Carter, and coworkers as discussed) that PPIs could inhibit EV release by disrupting a Hrs‐TSG101(or perhaps Alix‐TSG101) interaction mediated through ubiquitin. ILV formation and EV release could be assessed in the same cells under the previously reported conditions, however, this time, the endogenous TSG101 pool could be depleted and replaced with TSG101 with or without a C73A mutation. During PPI treatment, If ILV formation and EV (exosome) release are restored by the C73A TSG101 substitution, which precludes covalent binding of PPI to TSG101, then a role for a mono‐ubiquitinated Hrs (Alix)—TSG101 UEV interaction in ILV formation and EV release would be supported (assuming that the TSG101 C73A substitution itself does not preclude the interaction). If not, then endosomal alkalinization would be the presumptive mechanism for PPI inhibition of EV release as described earlier.

#### 2.2.5 NHEI, dimethylamiloride blocks EV release

As a counter‐regulatory mechanism for V‐H^+^‐ATPase, the Na^+^/H^+^ exchanger (NHE6‐NHE9) was reported to be a shunt pathway for protons in endosomes (Kondapalli et al., 2014; Nakamura et al., 2005; Nowak‐Lovato et al., 2010; Ohgaki et al., 2010; Prasad & Rao, 2015). Dimethylamiloride (DMA), a potent antagonist of the Na^+^/H^+^ exchanger (Teiwes & Toto, 2007), decreased basal EV release (Chalmin et al., 2010; Pironti et al., 2015; Savina et al., 2003). However, whether DMA decreased basal EV release by inhibiting the endosomal Na^+^/H^+^ exchanger(s), thus further reducing intraluminal pH, depended on whether DMA inhibited the specific NHE member(s) in endosomes. Moreover, not all investigators agreed that the Na^+^/H^+^ exchanger is a shunt pathway for protons, rather some proposed that it works in concert with V‐H^+^‐ATPase to acidify endosomes (Gekle et al., 1999; Milosavljevic et al., 2014). To confuse matters further, low micro‐environmental pH was associated with increased EV release from tumor cells (Parolini et al., 2009). Nevertheless, whether DMA may have increased or decreased intraluminal pH depending on the role of the Na^+^/H^+^ exchanger(s) in endosomes, in either case, one might speculate that the intraluminal pH of the early endosomes and MVB could have been sufficiently deviated from physiological levels to impair endosomal maturation and ILV formation, and hence, the release of exosomes. (Another possibility is that NHE is not necessarily an important regulator of MVB pH, but rather of Na^+^ or K^+^ concentrations, the counter‐ions, which may be a critical factor in ILV formation as proposed by Lawrence and colleagues (Lawrence et al., 2010). Interestingly, rather than being reduced, the number of MVBs were increased when NHE8 was knocked‐down by siRNA in HeLa cells suggesting a negative role for NHE8 on ILV formation or a positive role on ILV back fusion with the MVB‐limiting membrane (Lawrence et al., 2010). Impaired fusion of MVBs with lysosomes was a less likely explanation, because EGF degradation was increased in this study. But whether NHE8 knock‐down may have impaired fusion of MVBs with the plasma membrane for the release of exosomes was not explored.) Of course, an important factor potentially confounding these explanations is that DMA undoubtedly inhibited the sodium‐hydrogen exchanger in the plasma membrane. Although DMA was consistently reported to reduce the release of EVs (Chalmin et al., 2010; Pironti et al., 2015; Savina et al., 2003), the precise mechanisms do not seem to be entirely clear.

#### 2.2.6 Activation of multiple cellular receptors increase EV release

As mentioned earlier, if the predominant role of EVs is to impede viral infection, then increasing the release of EVs may prove beneficial. Although stimulated release of EVs has not been extensively and systematically investigated (Alonso et al., 2005; Pironti et al., 2015; Verweij et al., 2018), activation of several G‐protein‐coupled receptors (GPCRs) was recently found to increase EV release from a single cell type (trophoblast‐derived cells) (Conrad et al., 2020). It was speculated that, because there are as many as 800 GPCRs in the human genome, many of which can be expressed by one given cell‐type (Kroeze et al., 2003), perhaps each GPCR (or cohort of functionally related GPCRs) can affect EV release in a cell‐type‐specific manner containing a unique subset of cargo (e.g., RNAs, proteins, lipids) that, in turn, coordinates specific physiological responses among neighboring and distant cells (Conrad et al., 2020). (It also seems likely that some GPCRs might inhibit rather than stimulate EV release, although this possibility was not investigated.) In relation to SARs‐CoV‐2, it would be desirable to augment the release of EVs from cell‐type(s) generating EV cargo that is inhibitory of viral infection by activating the relevant cellular GPCR(s).

#### 2.2.7 Summary: Manipulation of EV release by PPIs, NHEIs, and G‐protein‐coupled receptors

If EVs facilitate SARs‐CoV‐2 pathogenesis, then by decreasing EV release, patient prognosis and clinical outcomes could be improved. PPIs were reported to reduce the release of EVs from tumor cells, presumably as a consequence of inhibiting vacuolar‐H^+^‐ATPase, which perturbs intraluminal pH within intracellular organelles of the endocytic pathway including multivesicular bodies where low pH may be critical to the formation of intraluminal vesicles (nascent exosomes). Although it seems likely that PPIs would also attenuate the release of EVs from nontransformed cells, this assumption has apparently not been tested. Other potential mechanisms for the reduction of EV release by PPIs include inhibiting the function of other proteins besides V‐H^+^‐ATPase in the MVB that are critical to ILV formation by covalently binding to thiol groups or by selectively disrupting the ESCRT machinery, which is important for their biogenesis. Although selective disruption of the ESCRT mechanism by PPIs seems less likely based on current knowledge as described earlier, the possibility apparently has not been formally tested. In addition to PPIs, dimethylamiloride was demonstrated to inhibit the release of EVs, but the underlying mechanism(s) also remain unclear. If EVs impede SARs‐CoV‐2 pathogenesis, then augmentation of EV release could improve patient prognosis and clinical outcomes. To this end, activation of GPCRs leading to increased EV release might be beneficial. Whether the net balance of EV action is facilitatory or inhibitory of SARs‐CoV‐2 pathogenesis may conceivably depend also on the stage of viral infection. Ultimately, it would be desirable to enhance the release of those EVs harboring cargo that impedes viral infection, while reducing the release of harmful EVs.

### 2.3 Viruses

#### 2.3.1 PPIs inhibit cellular release of some, but not all viruses

As mentioned earlier, several enveloped RNA viruses like HIV‐1 harness the ESCRT pathway for viral egress (Hurley & Cada, 2018). PPIs inhibited the budding of some (but not all) viruses hijacking the ESCRT machinery including Ebola (filoviridae family), Mayaro (togaviridae family), and the Epstein Barr virus (herpesviridae family) (Strickland et al., 2017; Watanabe et al., 2020). In light of their PPI‐sensitivity, these viruses were proposed to require the interaction of a ubiquitin moiety (e.g., a ubiquitin moiety in HIV‐1 Gag‐C terminal p6 protein) with the UEV ubiquitin‐binding domain of TSG101 for viral assembly and budding (Strickland et al., 2017; Watanabe et al., 2020). However, viruses that budded into the endoplasmic reticulum (ER), Dengue and Zika (flaviviridae family), were PPI‐resistant even though TSG101 and ESCRT‐III subunits were reported to be required for flaviviridae (Dengue and Japanese encephalitis) assembly on the ER membrane, and subsequent budding into the ER lumen (Tabata et al., 2016). The mechanism(s) for recruitment of TSG101 to the ER membrane by flaviviridae (Japanese encephalitis virus) involved PT(S)AP, but interestingly, not the UEV ubiquitin‐binding domain of TSG101 (Tabata et al., 2016). The latter observation was consistent with the failure of PPIs to inhibit flaviviridae viruses (Watanabe et al., 2020).

A major difference between SARs‐CoV‐2 and other enveloped RNA viruses like HIV‐1 is that biogenesis occurs in the endoplasmic reticulum‐Golgi intermediate compartment (ERGIC), and not on the plasma cell membrane (Hogue & Machamer, 2008; Schoeman & Fielding, 2019; Stertz et al., 2007). However, budding into the ERGIC apparatus involves the same membrane topology, and as such, requires host‐ or viral‐derived scission machinery (Rossman & Lamb, 2013). Whether SARs‐CoV‐2 co‐opts the ESCRT machinery for membrane scission in a manner analogous to HIV‐1 is presently unclear (Fujii et al., 2007; Schoeman & Fielding, 2019). If so, then PPIs could potentially inhibit SAR‐CoV‐2 budding into the ERGIC compartment. Alternatively, SARs‐CoV‐2 could engage TSG101 and the ESCRT machinery in a manner similar to flaviviridae through the PT(S)AP motif (alone), but independent of an interaction with an ubiquitin moiety and the UEV ubiquitin‐binding domain of TSG101 as in HIV‐1 (Tabata et al., 2016). However, the PT(S)AP motif important for TSG101 recruitment has not so far been identified in SARs‐CoV‐2 (Sobhy, 2020), but interestingly, nor was it found in flaviviridae in which the motif was critical to TSG101 recruitment (Tabata et al., 2016). Alternatively, SARs‐CoV‐2 could utilize viral‐coded protein(s) for budding and scission, for example, the SARs‐CoV‐2 envelope (E) protein analogous to influenza A matrix protein 2 (Nieva et al., 2012; Westerbeck & Machamer, 2015). In either case, though, SARs‐CoV‐2 would be PPI resistant.

Nevertheless, PPIs could also impede the life cycle of SARs‐CoV‐2 by inhibiting V‐H^+^‐ATPase (vide supra). Conceivably, increased luminal pH could disrupt SARs‐COV‐2 during endocytosis, thereby impeding cell entry, and/or in the ERGIC‐Golgi apparatus. However, the mechanisms for SARs‐CoV‐2 entry into cells are not conclusively established, and may vary by cell type. The mechanisms described to date include: (a) furin “preactivation” or “precleavage” of the spike protein S1/S2 junction during viral packaging in the ERGIC‐Golgi apparatus or secretory vesicles prior to egress from infected cells, thereby dissociating (or facilitating dissociation by subsequent proteases on target cells) of the spike protein S1 and S2 subunits necessary for S2‐mediated viral‐cell membrane fusion (Shang et al., 2020); (b) serine protease TMPRSS2 cleavage of the spike protein S1/S2 junction on the target cell plasma membrane (Bestle et al., 2020; Hoffmann, Kleine‐Weber, et al., 2020); and (c) endosomal cathepsin B/L protease cleavage of the spike protein S1 and S2 subunits leading to viral‐endosomal membrane fusion within the target cell (Qiu et al., 2006). Indeed, several investigators have demonstrated that inhibition of cathepsin B/L protease by increasing endosomal pH with bafilomycin, (hydroxy)chloriquine or ammonium chloride inhibited or attenuated CoV cell entry (Burkard et al., 2014; Milewska et al., 2018; Yang et al., 2004) including SARs‐CoV‐2 (Hoffmann, Kleine‐Weber, et al., 2020; Shang et al., 2020). There seems to be a redundancy of entry mechanisms, which conceivably may also work together. Alternatively, furin preactivation may be the dominant mechanism for cell entry in ACE2 expressing cells with low expression of TMPRSS2 and cathepsin B/L (Burkard et al., 2014; Shang et al., 2020). Finally, the spike protein of SARs‐CoV‐2 and its receptor, ACE2, are heavily glycosylated in the Golgi (Hogue & Machamer, 2008). Conceivably, glycosylation could be disrupted by PPIs through inhibition of the Golgi V‐H^+^‐ATPase (Demaurex et al., 1998; Kellokumpu, 2019; Maeda & Kinoshita, 2010; Scott & Gruenberg, 2011; Vincent et al., 2005) or by binding to sulfhydryl groups of the key enzymes, thereby inhibiting their function (Liu et al., 2013). Paradoxically, the avian infectious bronchitis CoV monomeric E protein was recently reported to increase (neutralize*)* Golgi pH, thereby protecting the S protein from premature cleavage and production of impaired or noninfectious virions (Westerbeck & Machamer, 2019). This activity of the CoV E protein apparently did not involve inherent channel activity, but rather depended on interaction with a host protein, for example, V‐H^+^‐ATPase, Na^+^/H^+^ exchanger, etc.

As noted earlier, (hydroxy)chloroquine also increases intraluminal pH of endocytic vesicles, but the evidence has not demonstrated efficacy in the treatment of COVID‐19 (Mahase, 2020; Torjesen, 2020). Nevertheless, PPIs and (hydroxy)chloroquine raise intraluminal pH through different mechanisms (i.e., V‐H^+^‐ATPase inhibition vs. H^+^ buffering, respectively). Conceivably, PPIs might be more efficient in raising intraluminal pH than (hydroxy)chloroquine due to their different mechanisms of action such that higher doses of (hydroxy)chloroquine might be required for therapeutic efficacy. However, the window of safety between a therapeutic and toxic dose may be narrow (Smit et al., 2020), and the overall safety profile of (hydroxyl)chloroquine is of additional concern (Juurlink, 2020).

Another possible reason for the ineffectiveness of (hydroxy)chloroquine in treating COVID‐19 relates to the mechanism(s) of SARs‐CoV‐2 cellular entry as described earlier. In cells where plasma membrane TMPRSS2 rather than endocytic vesicle cathepsin B/L proteases predominates, then agents that raise intraluminal pH of endocytic vesicles would likely be less effective or ineffective. Indeed, this concept was recently supported as it pertains to chloroquine and Calu‐3 cells, a human lung cancer epithelial cell line, although primary pulmonary epithelial cells were not tested (Hoffmann, Mosbauer, et al., 2020). Whether other ACE2 expressing cells, for example, neuronal cells that are susceptible to SARs‐CoV‐2 infection might rely less on TMPRSS2 and more on endocytic vesicle cathepsin B/L proteases for spike protein cleavage has to my knowledge not been explored (Song, 2020). Of relevance is a recent report of the profiling of 12,000 FDA‐approved small molecules or molecules in clinical development for other indications that revealed a PPI and inhibitors of cysteinyl cathepsins as potential drug candidates for treatment of SARs‐CoV‐2 supporting the current thesis (Riva et al., 2020). However, these results may be biased, because a Vero cell‐based screening assay was employed, in which the entry mechanism for SARs‐CoV‐2 is apparently different from pulmonary epithelium being more dependent upon endocytosis (Hoffmann, Kleine‐Weber, et al., 2020). Finally, another publication recently appeared, which showed that the PPI inhibitor, omeprazole, inhibited the cytopathic effect of SARs‐CoV‐2 in the colorectal cancer cell line Caco2 with an IC_50_ of 34 µM. Perhaps of greater promise is that at a concentration of 8 µM, omeprazole potentiated the cytopathic inhibitory effect of remdesivir by 10‐fold in this cell line (Bojkova et al., 2020). It would be interesting to determine whether similar promising results would be observed in human pulmonary epithelial and other cells (especially in primary cell culture) targeted by SARs‐CoV‐2.

#### 2.3.2 NHEI, hexamethylene amiloride, may ameliorate CoV pathogenesis

Full‐length CoV E protein or the 40 amino acid N‐terminal hydrophobic transmembrane domain of SARs‐CoV and other CoV viruses expressed channel activity in planar lipid bilayers (so‐called viroporin) (Wilson et al., 2004, 2006). The preferred cation conductance whether for Na^+^ or K^+^ depended on the taxonomic group of the coronavirus (Wilson et al., 2004, 2006). Channel activity was blocked by the Na^+^/H^+^ exchanger inhibitor, hexamethylene amiloride (HMA) reflecting its broad biological activity, but not by amiloride (a potent ENaC inhibitor). HMA also inhibited human CoV‐229E replication in cultured cells (Wilson et al., 2006). Torres and colleagues further demonstrated that the SARs‐CoV E protein N‐terminal hydrophobic transmembrane domain formed a homopentamer in lipid bi‐layers resulting in channel activity (Torres et al., 2006, 2007). Importantly, in mouse models of SARs‐CoV, a mutation in the E gene that inactivated channel activity decreased pulmonary edema formation, improved lung epithelial structure and function, promoted recovery from disease, and increased survival (Nieto‐Torres et al., 2014). Whether SARs‐CoV‐2 expresses a comparable E protein N‐terminal hydrophobic transmembrane domain that confers channel activity is presently unknown. However, the product of ORF3h was predicted to be a 40 amino acid protein with a single transmembrane domain that was 90% homologous to the corresponding protein encoded by SARs‐CoV as described earlier (Cagliani et al., 2020). Amiloride derivatives were reported to inhibit viroporins produced by other viruses, for example, HIV‐1 (Ewart et al., 2002).

Because NHE(s) contribute to pH regulation of organelles in the endocytic pathway, NHE inhibition might also disrupt the SARs‐CoV‐2 life cycle on this basis, analogous to PPIs. As mentioned earlier, however, it is not entirely clear whether NHEIs would increase or decrease pH of organelles in the endocytic pathway depending on whether the physiological role of the endosomal Na^+^/H^+^ exchanger is to shunt protons or abet V‐H^+^‐ATPase. An important consideration is that in one report, increasing Golgi pH actually facilitated rather than impeded avian infectious bronchitis CoV infection (Westerbeck & Machamer, 2019). Whether this finding would also apply to SARs‐COV‐2 remains to be determined. Conceivably, though, sufficient perturbation of intraluminal pH in either direction from physiological concentrations might disrupt the SARs‐CoV‐2 life cycle.

Whether the clinically available NHEIs designed to inhibit NHE1 and 3 (see *Safety*, below) can be repurposed depends on whether they would also inhibit the relevant NHEs expressed in endosomes (Faraone & Zhang‐James, 2013). Similarly, whether these clinically available NHEIs might inhibit viroporins is not clear. If not, then NHEIs would need to be developed for clinical use in ameliorating SARs‐CoV‐2 pathogenesis through these mechanisms.

#### 2.3.3 Summary: Can PPIs and NHEIs disrupt SARs‐CoV‐2 life cycle or ameliorate pathogenesis?

Proton pump inhibitors were shown to covalently bind to cysteine 73 in the ubiquitin‐binding pocket of TSG101 in ESCRT‐I, which precluded TSG101 accumulation at the plasma membrane, and subsequent HIV‐1 viral assembly and budding. The underlying mechanism was speculated to be through disruption of the interaction between the TSG101‐UEV domain with a ubiquitin moiety in HIV‐1 Gag‐C terminal p6 protein at the plasma cell membrane. However, PPIs did not similarly affect flaviviruses, which bud into the endoplasmic reticulum. Whether PPIs would inhibit SARs‐CoV‐2 assembly and budding in the ERGIC, to my knowledge has not been investigated. However, the PPI inhibitor omeprazole was recently reported to inhibit the cytopathic effect of SARs‐CoV‐2 in Caco2 cells, and to markedly potentiate the cytopathic inhibitory activity of remdesivir, but the underlying mechanism(s) were not elucidated. Both PPIs and NHEIs could disrupt SARs‐CoV‐2 life cycle by perturbing the pH of the endocytic vesicles, ERGIC and Golgi apparatus. They may be particularly effective in those cells in which the virus enters through endocytosis; in this case, elevated pH might inhibit cathepsin B/L protease activity and S1/S2 cleavage in the endocytic vesicle. It should be noted that in one study, raising Golgi pH facilitated rather than inhibited avian infectious bronchitis CoV virus production. By analogy to SARs‐CoV, NHEIs could potentially ameliorate pulmonary edema formation in SARs‐CoV‐2 infection by inhibiting channel activity conferred by the E protein. Further study is needed to test this possibility.

### 2.4 Safety

#### 2.4.1 PPIs

Proton pump inhibitors were generally considered to be safe for short‐term treatment of acute pathologies such as esophagitis and gastric ulcers. However, more recently they have been administered for long‐term treatment of chronic disease such as Barrett's esophagitis and hypersecretory states like Zollinger–Ellison syndrome, in order to ameliorate symptoms and prevent cancer. In postmarket analysis, chronic administration of PPIs was associated with increased risk for renal disease and electrolyte abnormalities (Makunts et al., 2019). If PPIs were found to be efficacious in treating COVID‐19, presumably they would be prescribed on a short‐term basis, thereby circumventing the safety concerns associated with chronic usage. However, PPIs will also raise gastric pH, which theoretically might render the intestinal tract more susceptible to SARs‐CoV‐2 infection (Zang et al., 2020; Zhou et al., 2017), and increase SARs‐CoV‐2 transfer across the intestinal epithelium (Almario et al., 2020). PPIs have also been shown to increase the risk of bacterial pneumonia, which can complicate the course of severe COVID‐19 (Fohl & Regal, 2011).

#### 2.4.2 NHEIs

In a Phase III prospective, randomized, double‐blind, placebo‐controlled trial, 11,590 patients with unstable angina or non–ST‐elevation myocardial infarction, or undergoing high‐risk percutaneous or surgical revascularization received either the NHE‐1 inhibitor, cariporide, or placebo. The intervention did not prevent myocardial cell necrosis in the setting of myocardial ischemia, the primary endpoint, but the safety profile of cariporide was favorable (Theroux et al., 2000). In a prospective, randomized, double‐blind, placebo‐controlled Phase II trial of 1,389 patients experiencing acute ST‐elevation myocardial infarction, eniporide, another NHE‐1 inhibitor, or placebo was administered. The intervention did not limit infarct size, the primary endpoint, but again, the safety profile of eniporide was good (Zeymer et al., 2001). Tenapanor. a NHE3 inhibitor, was recently approved for clinical use (Markham, 2019). As mentioned earlier, however, whether these NHEIs could inhibit other NHE member(s), for example, NHE6‐9 expressed in endosomes or viroporins is uncertain (Faraone & Zhang‐James, 2013). If not, then suitable NHE inhibitors would need development.

## 3 Key points and suggestions for future investigations

1. Extracellular vesicles were reported to be both facilitatory and inhibitory of viral pathogenesis. On the one hand, if the net effect is facilitatory in SARs‐CoV‐2 infection, then inhibition of EV release by PPIs or NHEIs should be beneficial. On the other, if the net effect is inhibitory, then augmentation of EV release by activation of G‐protein‐coupled receptor(s) could be therapeutic. Conceivably, whether EVs are facilitatory or inhibitory might also depend on the phase of the infection. Animal models of SARs‐CoV‐2 infection could be initially used to explore the potential impact of PPIs, NHEIs, and GPCR ligands on circulating EV concentrations and cargos in the context of disease progression.
2. PPIs were reported to inhibit EV release at least from tumor cells, a finding that needs to be substantiated in nontransformed cells. The mechanism(s) underlying the inhibition of EV release by PPIs is not completely understood. But, by blocking V‐H‐ATPase and raising intraluminal pH of organelles in the endocytic pathway, the biogenesis of intraluminal vesicles in multivesicular bodies (or earlier step(s) in the pathway), and the release of exosomes may be impaired. Besides V‐H^+^‐ATPase, PPIs could potentially inhibit the function of other proteins critical to ILV formation by covalently binding to thiol groups. Although perhaps less likely, PPIs might directly inhibit the ESCRT pathway, thereby impairing EV biogenesis, another possibility that could be initially tested in cultured cells.
3. The mechanism(s) underlying the inhibition of EV release by NHEIs is not completely understood either. But, the intraluminal pH of organelles in the endocytic pathway might be perturbed by blocking NHE, which again, could impair biogenesis of intraluminal vesicles in multivesicular bodies or disrupt earlier event(s) in the process, and the release of exosomes This mechanism of action could also be initially tested in cultured cells.
4. By perturbing intraluminal pH of organelles in the endocytic pathway, ERGIC and Golgi both PPIs and NHEIs could disrupt the SARs‐CoV‐2 life cycle. On the one hand, their efficacy through altering the pH of the endocytic pathway would be likely greatest in those cells that used this mechanism for viral entry. On the other hand, alteration of intraluminal pH in the ERGIC and Golgi could disrupt viral assembly and critical glycosylation events, respectively; however, one study showed that by increasing Golgi pH, avian infectious bronchitis CoV infection was facilitated. Although perhaps unlikely based on current knowledge, PPIs might conceivably disrupt the budding process of SARs‐CoV‐2 in the ERGIC system through inhibition of the ESCRT pathway. Supporting the potential use of PPIs in ameliorating SARs‐CoV‐2 pathogenesis are two recent investigations: one study identified a PPI as a potential drug candidate after a large‐scale profiling of small molecules, and the other showed that another PPI potentiated the cytopathic inhibitory activity of remdesivir by 10‐fold. Assuming that viroporins play a role in pulmonary edema formation by SARs‐CoV‐2, NHEIs could prove to be therapeutic through blocking these channels. Again, further investigations in cell culture and animal models of SARs‐CoV‐2 infection are needed to support or reject the hypothesis that PPIs or NHEIs might be therapeutic by directly impacting the viral life cycle. Importantly, whether the current NHEIs approved for use in humans would block the NHE member(s) in endosomes or viroporins is uncertain. If not, then relevant NHEIs would need to be developed for clinical use assuming that preclinical studies supported a potential therapeutic role for NHEIs in ameliorating SARs‐CoV‐2 infection.
5. On a final note, SARs‐CoV‐2 virus and extracellular vesicles converge upon the integration of several recent studies. First, the FYVE finger‐containing phosphoinositide kinase (PIKfyve kinase) inhibitor apilimod was identified in a large‐scale survey of potential therapeutics to inhibit SARs‐CoV‐2 replication in Vero cells with an EC_50_ of 23 nM (Riva et al., 2020). Second, apilimod was also found to inhibit SARs‐CoV‐2 entry into 293/hACE2 cells with an EC_50_ in the low nM range (Ou et al., 2020). Third, PIKfyve inhibition with apilimod (0.5 µM) or downregulation with siRNA increased the release of exosomes from PC‐3 cells (Hessvik et al., 2016). Thus, on the one hand, apilimod could mitigate SARs‐CoV‐2 infection by inhibiting cell entry. On the other hand, by increasing exosomes that potentially harbor cargo that facilitates infection, apilimod could negate or diminish its therapeutic potential in vivo. If so, by inhibiting EV release with concurrent administration of PPIs or NHEIs, the therapeutic efficacy of apilimod might be unmasked or enhanced.

## CONFLICT OF INTEREST

No conflicts of interest, financial or otherwise, are declared by the author.
